# Reduced intraepithelial corneal nerve density and sensitivity accompany desiccating stress and aging in C57BL/6 mice

**DOI:** 10.1016/j.exer.2018.01.024

**Published:** 2018-01-31

**Authors:** Mary Ann Stepp, Sonali Pal-Ghosh, Gauri Tadvalkar, Alexa Williams, Stephen C. Pflugfelder, Cintia S. de Paiva

**Affiliations:** a Department of Anatomy and Regenerative Biology, The George Washington University School of Medicine and Health Sciences, Washington DC, USA; b Department of Ophthalmology, The George Washington University School of Medicine and Health Sciences, Washington DC, USA; c Department of Ophthalmology, Ocular Surface Center, Cullen Eye Institute, Baylor College of Medicine, Houston, TX, USA

**Keywords:** Cornea, Nerves, Desiccating stress, Dry eye disease, Mouse

## Abstract

Dry Eye disease causes discomfort and pain in millions of patients. Using a mouse acute desiccating stress (DS) model we show that DS induces a reduction in intraepithelial corneal nerve (ICN) density, corneal sensitivity, and apical extension of the intraepithelial nerve terminals (INTs) that branch from the subbasal nerves (SBNs). Topical application of 0.02% Mitomycin C (MMC) or vehicle alone has no impact on the overall loss of axon density due to acute DS. Chronic dry eye, which develops progressively as C57BL/6 mice age, is accompanied by significant loss of the ICNs and corneal sensitivity between 2 and 24 months of age. QPCR studies show that mRNAs for several proteins that regulate axon growth and extension are reduced in corneal epithelial cells by 24 months of age but those that regulate phagocytosis and autophagy are not altered. Taken together, these data demonstrate that dry eye disease is accompanied by alterations in intraepithelial sensory nerve morphology and function and by reduced expression in corneal epithelial cells of mRNAs encoding genes mediating axon extension.

## 1. Introduction

The cornea is innervated by a dense collection of sensory axons termed intraepithelial corneal nerves (ICNs) that consist of two components: subbasal nerves (SBNs) ensheathed by corneal epithelial basal cells and intraepithelial nerve terminals (INTs) which branch from the SBNs and extend perpendicularly towards the apical squames. While sympathetic nerves have been reported by some groups in the normal mouse cornea (Ivanusic et al., 2013; Marfurt and Ellis, 1993), others have been able to identify sympathetic nerves in the mouse cornea only after herpes simplex infection (Yun et al., 2016). The ICNs function as thermoreceptors and nociceptors and induce blinking and the spreading of tears over the ocular surface (Gonzalez-Gonzalez et al., 2017). The nerve cell bodies for the ICNs are located in the trigeminal ganglion (Lopez de Armentia et al., 2000). When damage to the trigeminal ganglion occurs or majority of the ICNs are severed, corneal sensitivity is lost, the corneal epithelium erodes, stroma becomes cloudy, and epithelial cells cease proliferating (Ferrari et al., 2011, 2013; Yamaguchi et al., 2013); ICNs are vital for maintaining a healthy ocular surface.

Advances in imaging allow the acquisition of high-resolution images of the ICNs on mouse corneas using confocal microscopy (Reichard et al., 2016; Stepp et al., 2017); similarly, advances in vivo confocal microscopy permit the study of the ICNs in human corneas (Patel and McGhee, 2009; Stachs et al., 2007). Altered morphology and numbers of the corneal sensory nerves have been reported by clinicians studying small fiber neuropathy and in diseases affecting the central nervous system including Multiple Sclerosis, Parkinson's Disease, and Fibromyalgia (Cruzat et al., 2017). In vivo corneal confocal imaging has been proposed as a method to diagnose the early stages of these conditions and follow the responses of patients to treatment.

The ICNs have been implicated in the discomfort associated with dry eye. Pain can be caused by increased friction between the eyelid and cornea due to reduced tear volume or quality and is exacerbated by inflammation (Goyal and Hamrah, 2016; Rosenthal and Borsook, 2016). The discomfort associated with dry eye disease has been proposed to represent, in some patients, ocular small fiber neuropathy (Andersen et al., 2017).

Advances in our understanding of dry eye disease have been made using both chronic and acute mouse models to study the disease and it's progression (Pflugfelder and de Paiva, 2017). Genetically engineered mice have been characterized as models for chronic forms of dry eye; these include the Aire mice (Chen et al., 2017; Yeh et al., 2009) and Langerin (CD207) deficient mice (Choi et al., 2017). Topical application of benzalkonium chloride to the ocular surface of genetically normal mice has been shown to induce dry eye (Sarkar et al., 2012). Allowing C57BL/6 mice to age is associated with development of chronic dry eye disease symptoms (McClellan et al., 2014) similar to those seen in young mice when subjected to treatments that induce dry eye disease acutely (Dursun et al., 2002). Sensory axon density has been reported to decrease in C57BL/6 mice (Reichard et al., 2016). A recent study shows that sensory axon density is reduced in a surgically induced chronic dry eye model where the lacrimal gland is removed (Yamazaki et al., 2017).

In the current study, we assess the impact of acute DS and aging on ICN density, thickness, and sensitivity as well as the ability of ICN axons to extend apically towards the epithelial surface. In addition, we assess the impact of the topical application of 0.02% Mitomycin C or vehicle alone (PBS) on axon density in C57BL/6 mice subjected to acute DS. Topical application of MMC (0.02%) is used clinically in ophthalmology during glaucoma and refractive surgical procedures to reduce scarring (Abraham et al., 2007; Santhiago et al., 2012; Shalaby et al., 2009). Using a BALB/c mouse model for recurrent epithelial erosions, we found that two topical applications of MMC (0.02%) delivered 3 and 7 days after 1.5 mm debridement wounding significantly improved resolution of erosions and increased axon density (Pal-Ghosh et al., 2016). The impact of topical MMC treatment on ICN density in dry eye disease has not been studied. Given the millions of patients who have dry eye disease and the numerous ophthalmic uses of topically applied MMC, here we also evaluate the impact of MMC on axon density in C57BL/6 mice subjected to acute DS.

## 2. Materials and methods

### 2.1. Animals

All studies performed were approved by The Institutional Animal Care and Use Committees at Baylor College of Medicine and comply with the ARVO Statement for the Use of Animals in Vision Research. Female C57BL/6 mice aged 6–8 weeks old were purchased from Jackson Laboratories (Bar Harbor, ME). DS was induced by subcutaneous injection of scopolamine hydrobromide (0.5 mg/0.2 ml; Sigma-Aldrich, St. Louis), QID (08:00, 12:00, 14:00, and 17:00 h), for 3, 5 or 10 consecutive days in 6–8 week old female C57BL/6 mice and euthanized at the end of each variable (DS3, DS5 and DS10), as previously published (de Paiva et al., 2009; de Paiva et al., 2006a; de Paiva et al., 2006b). Mice were placed in a cage with a perforated plastic screen on one side to allow airflow from a fan placed six inches in front of it for 16 h/day. Room humidity was maintained at 30–35%. Control mice were maintained in a non-stressed (NS) environment containing 50–75% relative humidity without exposure to forced air. Since dry eye is more prevalent in women and male mice do not respond well to desiccation, only female mice were used (Gao et al., 2015; Schaumberg et al., 2003, 2009).

### 2.2. Topical treatment regimen

Mice (DS5 and DS10) received one topical application of Mitomycin-C (0.02%), 2µl/eye, Green Park Pharmacy, Houston, TX) or vehicle (balanced salt solution, BSS, Alcon, Fort Worth, TX) at d1 and d3 after initiation of DS.

### 2.3. Corneal mechanical sensitivity

Corneal sensitivity was measured under a surgical loupe using a modified Cochet-Bonnet filament in the right eye only of each mouse. The standard Cochet-Bonnet nylon filament has a 0.12 mm diameter; we used a 9–0 nylon that has 0.03 mm diameter to compensate for smaller and thinner corneas in mice as compared to humans (Yamazaki et al., 2017). The nylon monofilament was cut in different lengths (1.0, 1.5, 2.0, 2.5, 3.0, 4.0 cm). While holding the animal, the nylon filament was applied to the cornea and a positive response was indicated by a clear stimulus–evoked blink and retraction of the eye into the ocular orbit. The central cornea was tested six times at each filament length. The response was considered negative when no blink was elicited by the monofilament touch. A positive response was considered when the animal blinked more than or equal to 50% the number of times tested. If no blink response could be elicited at a monofilament length of 1.0 cm, corneal sensitivity was recorded as 0.

### 2.4. Aging study

Naturally aged C57BL/6 mice of both sexes were maintained in specific pathogen free vivarium and used at 24 months of age. Only mice free of visible cornea pathology were used. Male and female mice were used unless otherwise indicated.

### 2.5. Evaluation of corneal irregularity

Corneal surface irregularity was assessed in young and aged C57BL/6 mice (n = 12 animals/group) as previously published (De Paiva et al., 2011; De Paiva et al., 2011; McClellan et al., 2014; de Paiva et al., 2009; de Paiva et al., 2006a). Briefly, reflected images of a white ring from the fiber-optic ring illuminator of the stereoscopic zoom microscope (SMZ 1500; Nikon) were taken immediately after euthanasia. This ring light is firmly attached and surrounds the bottom of the microscope objective. Because the illumination path is nearly coincident with the optical axis of the microscope, the viewing area is evenly illuminated and nearly shadowless. The projected ring light will reflect off a wet surface and the regularity of the reflected ring depends on the surface smoothness. The smoothness of the reflected rings was graded in digital images by a masked observer. The projected ring was divided into four quadrants of 3 clock hours each. The corneal irregularity severity score was calculated using a 5-point scale based on the number of distorted quadrants in the reflected ring: 0, no distortion; 1, distortion in one quadrant of the ring (3 clock hours); 2, distortion in two quadrants (6 clock hours); 3, distortion in three quadrants (9 clock hours); 4, distortion in all four quadrants (12 clock hours); and 5, severe distortion, in which no ring could be recognized. Corneal irregularity severity scores were obtained from both eyes of 12 young and 12 old mice.

### 2.6. Immunofluorescence

Fixing and staining of mouse corneas for identification of the intraepithelial nerve terminals has been described previously (Pal-Ghosh et al., 2016). Corneas were incubated with βIII tubulin (Biolegend, #801201) and/or ki67 (Abcam, # ab16667), at 4 °C.

### 2.7. Microscopy

Confocal microscopy was performed at the GW Nanofabrication and Imaging Center at the George Washington University Medical Center. For Sholl (25× magnification) and Thickness (40× magnification) analysis, images were acquired using the Zeiss Cell Observer Z1 spinning disk confocal microscope (Carl Zeiss, Inc., Thornwood, NY, USA), equipped with ASI MS-2000 (Applied Scientific Instrumentation, Eugene, OR, USA) scanning stage with z-galvo motor, and Yokogawa CSU-X1 spinning disk. A multi-immersion 25×/0.8 and 40×/1.4 objective lens, LCI Plan-Neofluor, was used for imaging, with oil immersion. Evolve Delta (Photometrics, Tucson, AZ, USA) 512 × 512 EM-CCD camera was used as detector (80-msec exposure time). A diode laser emitting at 568 nm was used for excitation (54% power). Zen Blue software (Carl Zeiss, Inc.) was used to acquire the images, fuse the adjacent tiles, and produce maximum intensity projections. The adjacent image tiles were captured with overlap to ensure proper tiling. All images were acquired using the same intensity settings. Sholl analysis was performed using ImageJ as described previously (Pajoohesh-Ganji et al., 2015).

For high-resolution immunofluorescence imaging, a confocal laser-scanning microscope (Zeiss 710) equipped with a krypton-argon laser was used to image the localization Alexa Fluor 594 (568 nm excitation; 605/32 emission filter). Optical sections (*z* = 0.5 µm or 1 µm) were acquired sequentially with a 63× objective lens. For quantification of intraepithelial nerve terminals, 3D images were rotated to generate cross section views using Volocity software (Version 6.3, Perkin Elmer) and images were presented as cross sections projected through the length of the acquired image (135 µm). Pixel intensity data for apical and basally projecting nerve fibers were obtained as described previously (Pal-Ghosh et al., 2017) for no fewer than 4 corneas per variable.

### 2.8. QPCR

For quantitative polymerase chain reaction (QPCR) studies, epithelium was scraped using a dulled blade and frozen immediately. Four corneas per sample and at least 3 samples per time point were used for the QPCR studies. RNA was extracted using a QIAGEN RNeasy Plus Micro RNA isolation kit (Qiagen) following the manufacturer's protocol. After isolation, the concentration of RNA was quantified using a NanoDrop^®^ ND-2000 Spectrophotometer (Thermo Scientific, Wilmington, DE) and stored at −80 °C until used. QPCR was performed using a Bio-Rad CFX384 Real-Time PCR detection system. The primers used were ordered from Bio Rad, unless otherwise specified: Bec1(qMmuCID0005981), LC3 (qMmuCED0045817), LAMP1 (qMmuCID0027030), LAMP2 (qMmuCID0011408), CXCL1(qMmuCED0047655), BDNF(qMmuCED0050333), NTN1(Qiagen #QT00128478), DCC(Qiagen #QT00135100), Unc5b(Qiagen #QT00167846), Efna4(Qiagen #QT00100681), Efna5(Qiagen #QT00116494), Rgma (Qiagen #QT00310583) and GAPDH (qMmuCED0027497). QPCR data was normalized against GAPDH.

### 2.9. Statistical analyses

Quantitative data are presented as mean ± standard error of the mean. All data were analyzed using one-way ANOVA; when standard deviations were significantly different from one another as determined by Barlett's test, the Kruskal-Wallis Multiple Comparisons test was performed as specified by Graphpad Prism, Version 6 (GraphPad Software, Inc. San Diego, CA). All Statistical calculations were performed using Prism, (GraphPad Software, Inc. San Diego, CA). A **p** value < 0.05 was considered statistically significant.

## 3. Results

### 3.1. Axon density and thickness are reduced in response to acute DS

**Fig. 1:**
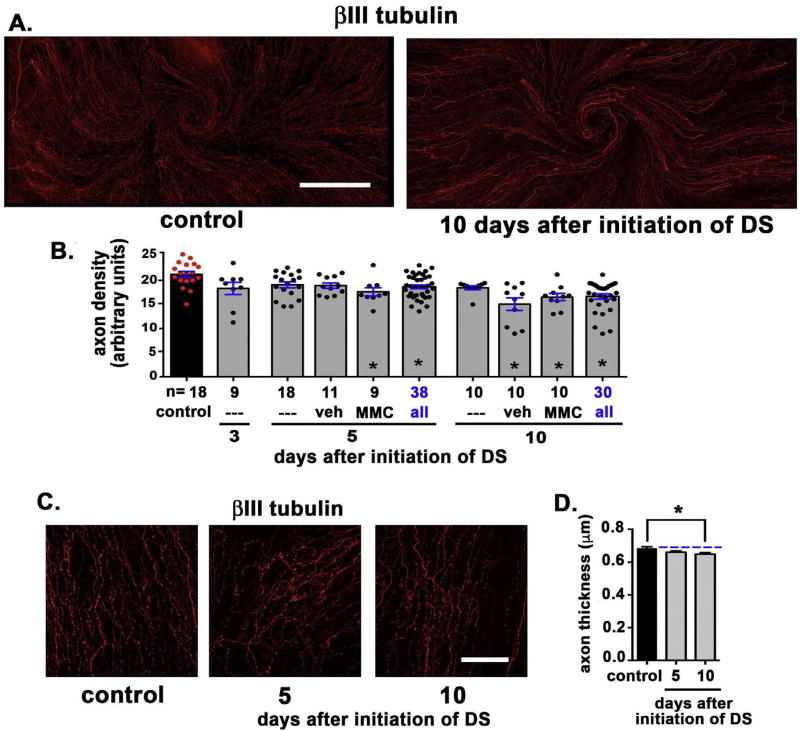
Axon density and thickness are reduced in response to acute DS **A.** Representative images of a control and an acute DS cornea from d10 are presented. The ICNs have been visualized using an antibody against βIII tubulin. A minimum of 5 corneas for each variable were assessed. **B.** Images including those presented in **1A** were used to quantify the axon density of the ICNs using Sholl analysis. The total number of corneas assessed (n) are indicated; both eyes of each animal were used. Each red or black circle represents the axon density of one cornea. Axon density decreased significantly relative to controls at d5 after MMC treatment and at d10 after both vehicle (veh) and MMC treatment. **D**. Representative images of corneas used to measure mean axon thickness are presented. **E**. Mean axon thickness was quantified and is presented. Axon thickness is reduced at d5 and d10 compared to controls. Bar in A = 500 µm; bar in D = 100 µm. (For interpretation of the references to colour in this figure legend, the reader is referred to the Web version of this article.)

DS was induced acutely in 8-week-old C57BL/6 mice as described previously using cholinergic receptor blockade (scopolamine) and a low humidity drafty environment (De Paiva et al., 2007). MMC (0.02%) or vehicle (PBS) was applied topically at d1 and d3 after initiation of scopolamine treatment and placing mice in blower cages. Corneas from mice euthanized at d3 had not been treated with MMC or vehicle. Mice maintained under standard housing conditions were used as controls. Fig. 1A shows representative images of a control and an acute DS cornea from d10. The number of control and DS corneas evaluated at days 3, 5, and 10 after initiation of DS are indicated in the graph in Fig. 1B. Axon density for each cornea is shown either as red (control) or black (DS) filled circles at 3, 5, and 10 days after initiation of DS. Data for the three d5 and d10 subgroups [no treatment (—), vehicle (veh), and MMC-treated (MMC)] are presented individually and after being combined to increase the statistical power of the assessments (blue text).

Relative to control mice, axon density decreased significantly at d5 after MMC treatment and at d10 after vehicle (veh) and MMC treatment. When axon density data for the three d5 and d10 subgroups were compared to one another by the Kruskal Wallis Multiple Comparisons test, no significant differences were seen between subgroups at either time point. Axon density data for the subgroups were combined into a single group that included 38 (d5) and 30 (d10) mice. Relative to controls, the combined data shows that axon density is significantly reduced by d5 and remains decreased at d10.

Next, we assessed mean axon thickness. Representative images from these assessments are presented in Fig. 1D and data quantitated in Fig. 1E. The images used for these assessments are single en face confocal images acquired using a 63× oil immersion objective through the center of the cornea. Mean axon thickness is reduced at d5 and d10 compared to controls. Data from veh and MMC treated corneas are not included in this and subsequent assessments.

**Fig. 2:**
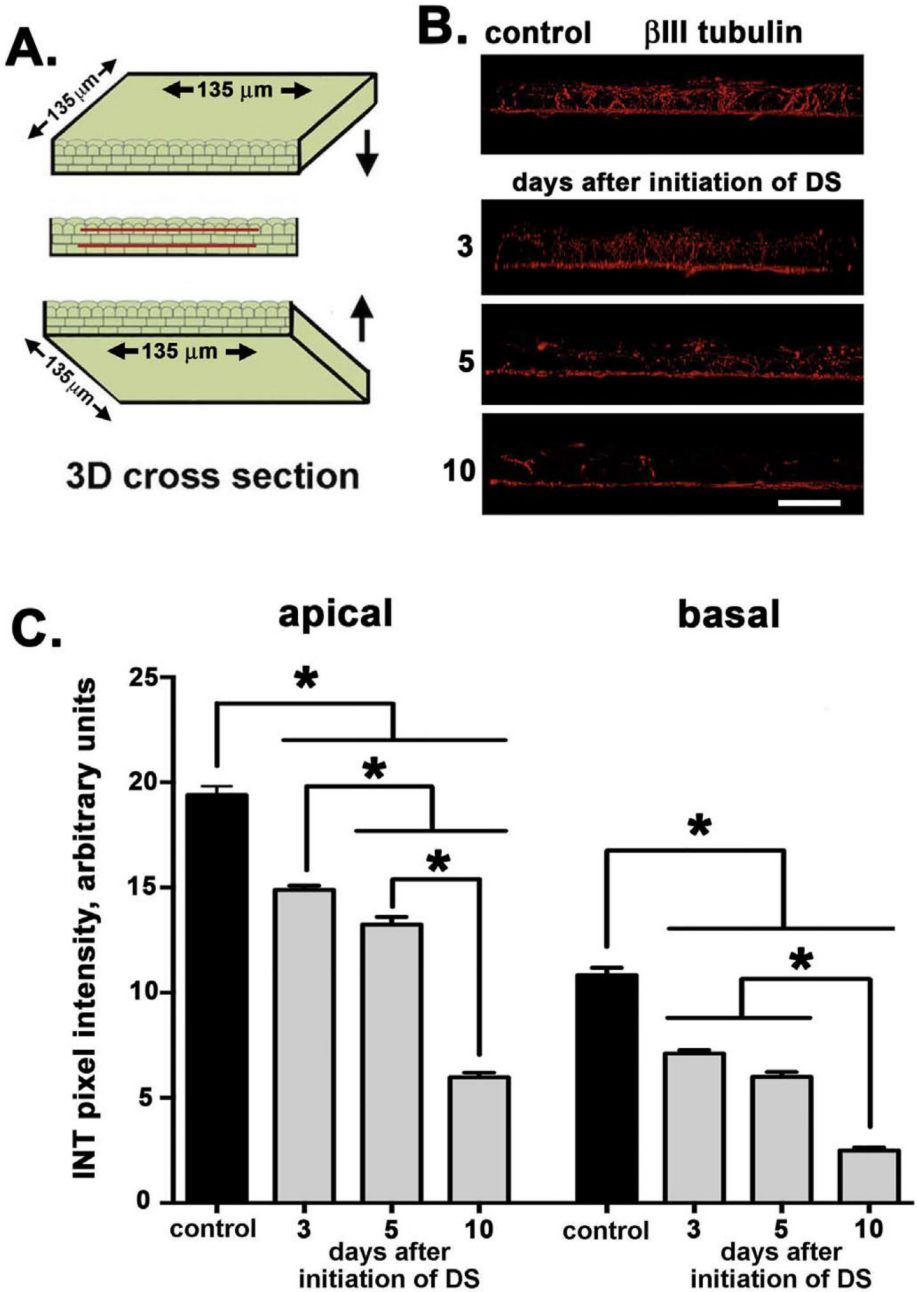
Apical axon extension of intraepithelial nerve terminals (INTs) is reduced in response to acute DS **A**. High-resolution confocal image stacks were obtained to generate 3D images; images were rotated to generate cross sectional views through 135 µm of cornea tissue as represented schematically. **B**. Representative images taken for assessment of axon terminal apical extension are presented. **C.** INT extension apically decreases within both the apical and basal sites assessed at d3 and continues to decrease through d10 after induction of acute DS. By d10, there is a 70–80% reduction in the extension of the subbasal nerves towards the apical aspect of the cornea. Data were obtained from 3 to 5 corneas for each time point assessed. Bar in B = 25 µm.

The method used to quantify axon density uses flattened projection confocal image stacks and does not permit assessment of the intraepithelial nerve terminals (INTs). To look at apical INT extension, we obtained high-resolution confocal image stacks from 3 to 5 corneas for each variable, generated 3D images, and rotated them to generate cross sectional views projecting through 135 µm of cornea tissue (see cartoon Fig. 2A). INT density was assessed by quantifying pixel intensity values across two zones parallel to the basal cell layer; one line was placed above the subbasal nerve mat (basal) and the other below the apical most cell layers (apical). Fig. 2B shows representative images taken for assessment of axon thickness. Quantitative data are presented in Fig. 2C. The density of the INTs decreases within both the basal and apical zones at d3 after induction of acute DS; the reduction in apical projection of axons persists and becomes greater at d5 and, by d10, there is a 70–80% reduction in the extension of the subbasal nerves towards the apical-most region of the corneal epithelium. Next, ki67 staining was used to assess if there were differences in corneal epithelial cell proliferation between control and DS corneas. No significant differences in the numbers of ki67 + cells per field were observed at any time point evaluated (data not shown).

### 3.2. Corneal sensitivity, assessed using corneal touch threshold, is lost within 3 days after induction acute DS

**Fig. 3:**
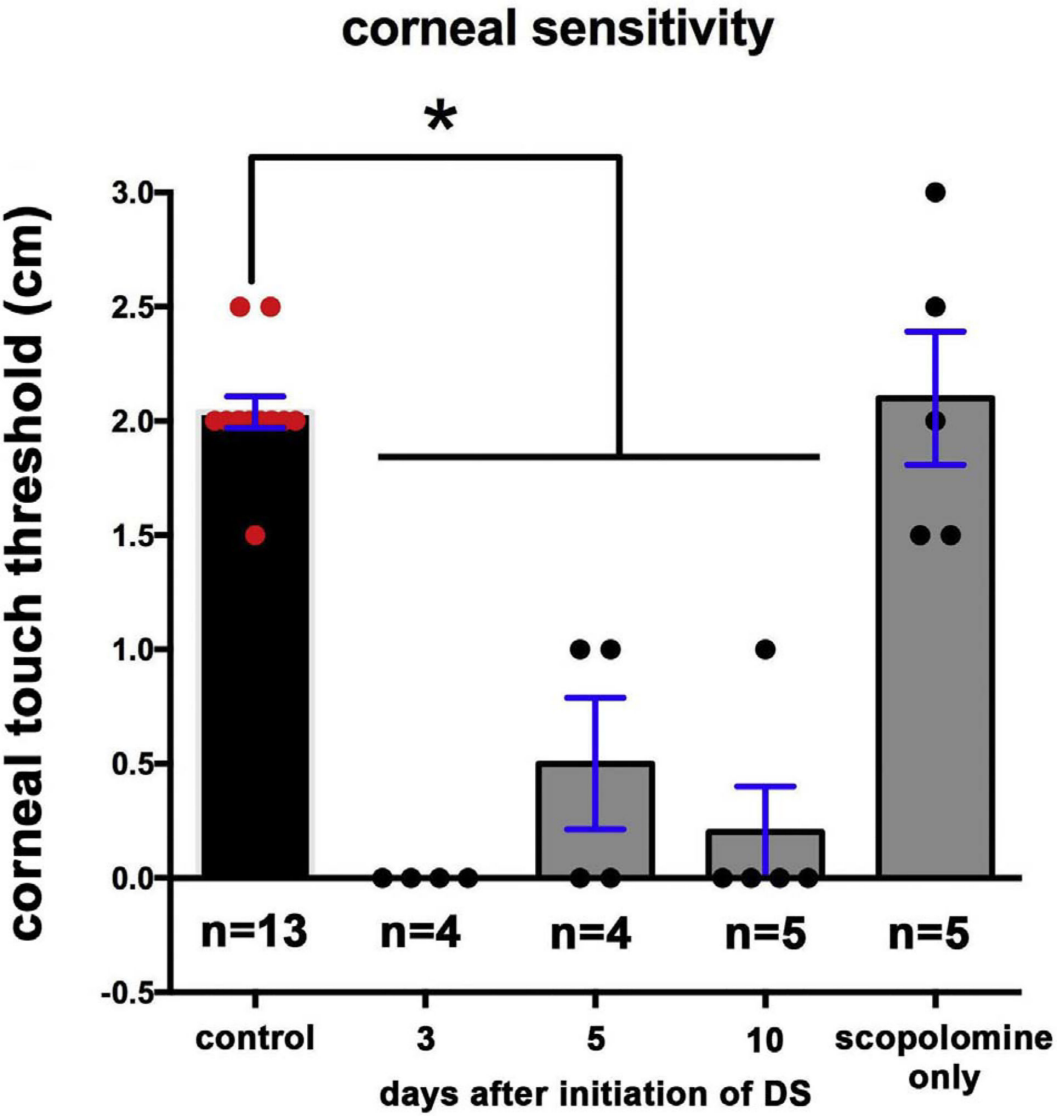
Acute DS decreases corneal touch threshold To determine whether the differences in ICN morphology impact the function of the corneal sensory nerves, corneal sensitivity was assessed by quantifying the pressure required to induce the blink response. Assessments were made on right eyes only. Within 3d after induction of DS, corneal touch threshold is significantly reduced and remains reduced. Control corneas of mice treated with scopolamine but not placed under airflow in low humidity show corneal touch thresholds similar to controls.

While the overall decrease in axon density shown in Fig. 1C at d10 is less than 20%, the projection of those axons apically to the ocular surface is reduced by 70–80% as shown in Fig. 2C. Axons that terminate beneath the apical-most squames depolarize first in response to cold or mechanical stimuli compared to axons that terminate deeper in the epithelium within the basal cell layer. To determine whether the morphological differences seen in the sensory nerves impact nerve function, we next assessed sensitivity by determining the corneal touch threshold using a modification of the Cochet-Bonnet aesthesiometer (Rao et al., 2010) as described in the methods section. This assessment involves touching a thin nylon filament of varying lengths to the corneal surface and evaluating the blink response. Data are presented in Fig. 3 and show that the corneal touch threshold was eliminated at d3 and remained suppressed through d10 after initiation of DS. Mice injected with scopolamine but not subjected to airflow or low humidity show no reduction in corneal touch threshold. In summary, by d3 after DS is initiated, corneal touch threshold is eliminated even though the reduction in apical extension of the nerve terminals at d3 is less than 30% and there is no significant reduction in overall axon density.

### 3.3. The ocular surface of aged mice shows increased roughness and reduced intraepithelial corneal axon density

**Fig. 4:**
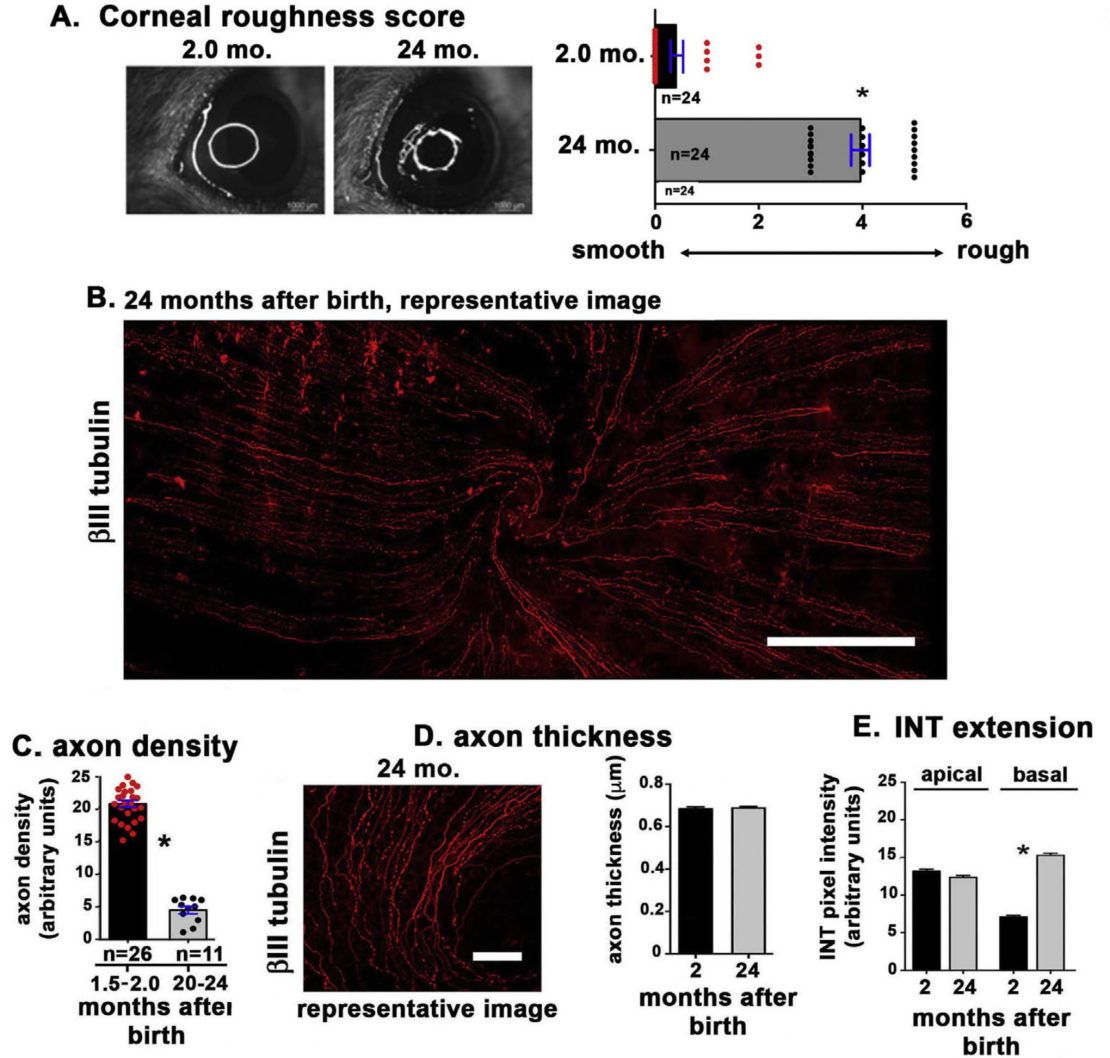
Aged mice show increased ocular surface irregularity and altered corneal sensory nerve morphology **A.** Ocular surface smoothness was assessed by quantifying the light relected off the ocular surface when illuminated with a fiber-optc ring light as described in the methods section. Data show that the ocular surface of 24 month old mice is significantly rougher than that of 2 month old mice. **B.** To determine whether axon density changes with age, we assessed axon density in corneas from mice that were 1.5 to 2.0 months old and compared it to mice that were 20 to 24 months old. A representative image from a 24-month-old mouse is presented. **C**: Quantitation of axon density data from 1.5 to 2.0 and 20 to 24-month-old mice show that axon density decreases significantly by ~75% by 20 to 24 months of age. Both left and right corneas were used for these studies; n values refer to the total number of corneas assessed. **D**: Representative images used to quantify axon thickness and the quantification of axon thickness obtained from analysis of 3 to 5 corneas per variable are presented for 2 and 24 month old mice. Data indicate axon thickness does not decrease as mice age. **E.** The extent of apical extension of the nerve terminals was next assessed in 2 and 24 month old mice using the method described in Fig. 2. Nerve terminal apical extension is not impaired with aging; it increases significantly within the basal cell layer. Bar in B = 500 µm; bar in D = 100 µm.

We previously reported that the corneas of 15 to 24-month-old female C57BL/6 mice show evidence of dry eye disease with increased Oregon Green dextran (OGD) corneal staining, reduced conjunctival goblet cell density and corneal surface irregularity noted in reflected rings (McClellan et al., 2014; Volpe et al., 2016). To confirm our previous findings, we show corneal surface irregularity scores in 2 and 24 month-old mice (Fig. 4A). Data confirm an increase in the corneal surface irregularity with aging consistent with previous studies. To determine whether mice also show changes in their sensory nerves with aging, we compared axon density of 6 to 8 week old mice to that of 20 to 24 months. While data from female mice are presented, similar results were obtained for male mice (data not shown). The representative image in Fig. 4B shows a cornea from a 24-month-old mouse; when compared to data shown in Fig. 1A for the 8-week-old control mouse cornea, fewer axons are seen which appear thinner and are often disrupted. Quantitation of axon density data from 1.5 to 2.0 and 20 to 24 month-old mice are presented in Fig. 4C and show that axon density decreases by ~75% at 20 to 24 months of age (from 21 to 5). The graph shows axon density values for each cornea assessed. When we assessed axon thickness (Fig. 4D), we found no change with aging. The ability of the intraepithelial axon terminals to target to the apical most cell layers was also assessed (Fig. 4E); we found axon extension was retained with aging and increased within the basal cell layer. Although much less abundant, the axons that are retained at 24 months are capable of targeting to the apical surface of the cornea.

### 3.4. By 24 months corneal sensitivity is lost in the majority of corneas assessed and cell proliferation is reduced

**Fig. 5:**
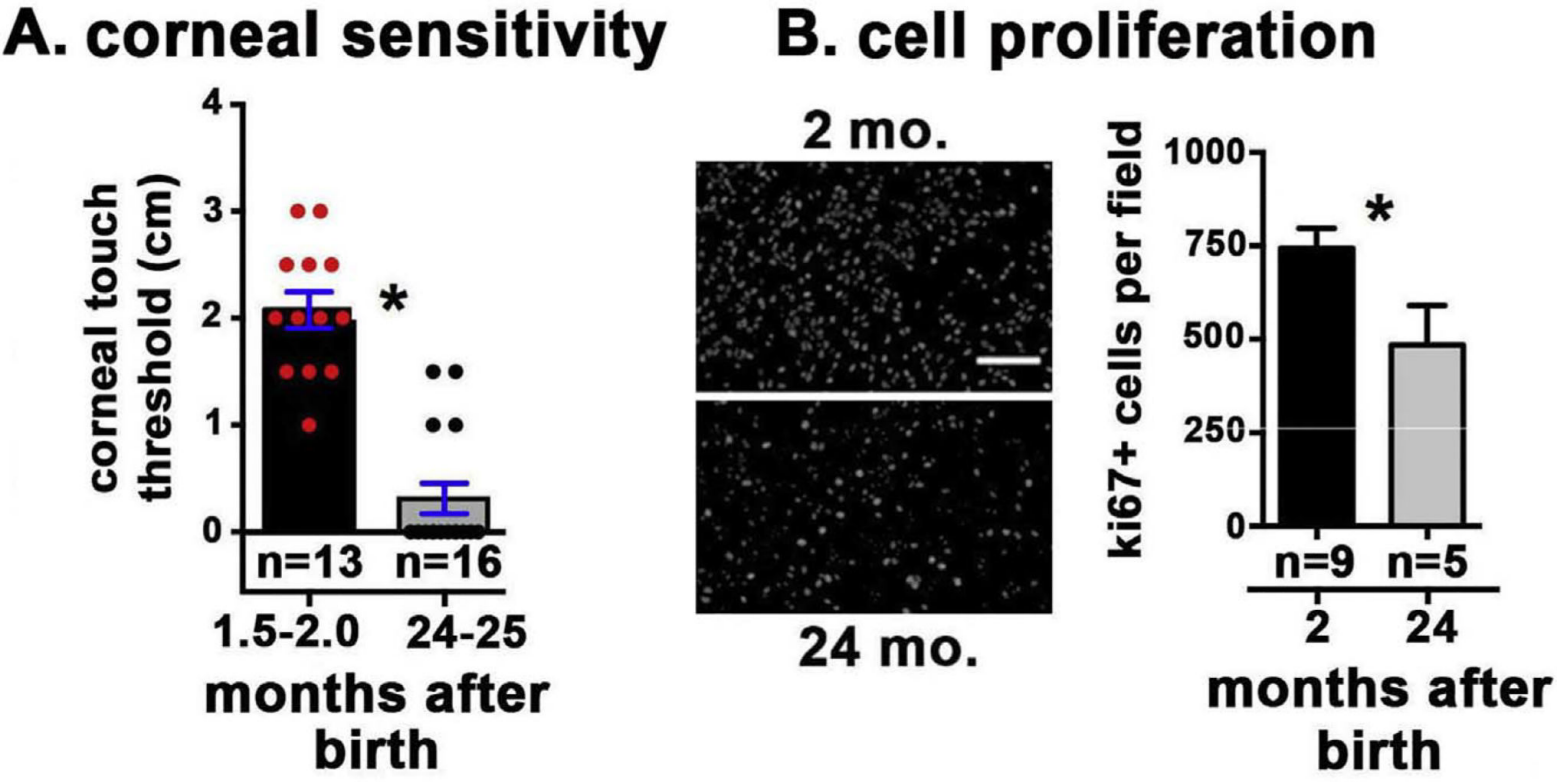
Aged mice show reduced corneal sensory nerve function and corneal epithelial cell proliferation **A.** Corneal sensitivity was assessed by quantifying corneal touch threshold. Sensitivity to touch was decreased significantly in 24-month-old mice compared to 2-month-old mice. The n values indicated refer to individual corneas assessed; since only right eyes were evaluated, for this assay, n also equals the numbers of mice used. **B.** Cell proliferation was quantified by counting the number of ki67 + cells per field in 3 to 5 corneas each of 2 and 24 month old mice. Four corneas were assessed per variable. Representative en face images of flat mounted corneas are shown on the left and quantification is shown on the right. Data show a significant decrease in cell proliferation in the 24-month-old mice. Bar in B = 40 µm.

We next assessed corneal sensitivity in control and 24-month-old mice Fig. 5A. The 24-month-old mice showed a significant loss of sensitivity as assessed by corneal touch threshold, and 12 of the 16 corneas did not blink in response to the nylon filament. The impact of the loss of axon density and sensitivity with aging on corneal epithelial cell proliferation was also assessed. Representative en face ki67 images and quantitation of ki67 + cells per field are shown in Fig. 5B. We observe a significant decrease in corneal epithelial cell proliferation between 1.5 and 2.0 month and 24 month-old mice. While the axons retained at 24 months targeted to the apical surface, they did not support normal sensation or corneal epithelial cell proliferation.

### 3.5. Age-induced dry eye is accompanied by reduced expression of the mRNAs encoding proteins that mediate axon growth and targeting and increased expression of an mRNA encoding a protein that represses axon growth

**Fig. 6:**
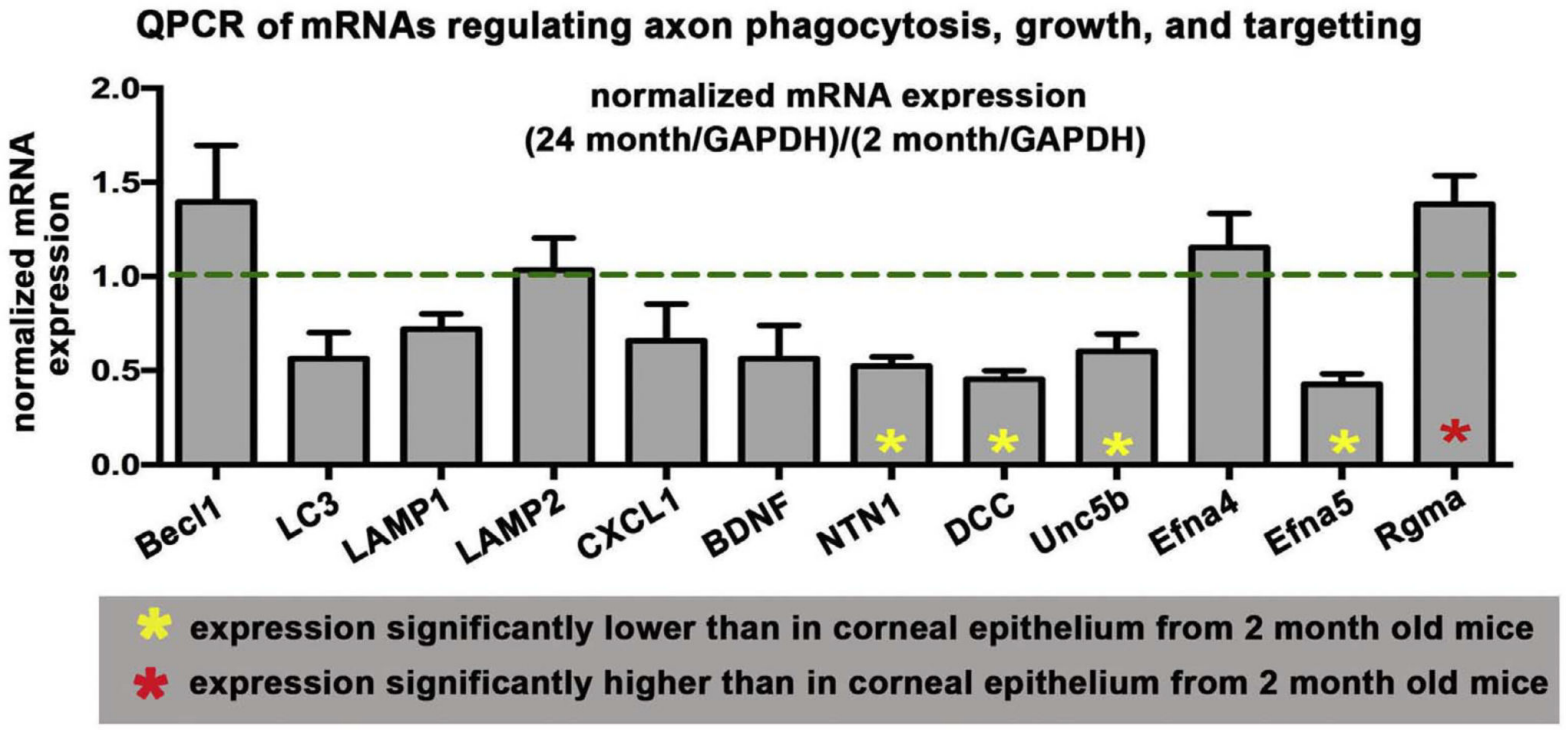
Expression of mRNAs for proteins that mediate axon targeting are altered in mRNA isolated from corneal epithelial cells of aging mice but those mediating phagocytosis and autophagy do not change QPCR studies were performed on mRNA isolated from the corneal epithelium of young (1.5 to 2.0) and old (20 to 24 month) mice. Data for 24 month-old-mice after normalization against mRNA expression relative to 1.5 to 2.0 month old mice are shown. Asterisks within bars indicate significant differences in RNA expression relative to RNA isolated from 1.5 to 2.0 month old mice. There were no significant differences seen with aging in expression of mRNAs that are activated during phagocytosis and autophagy (Beclin1, LC3, LAMP1, and LAMP2). However, mRNA expression for several proteins that enhance axon targeting were significantly reduced (yellow asterisks) at 24 months including netrin1 and it's receptors DCC, Unc5b and Efna5. Repulsive guidance molecule A (Rgma) was significantly increased (red asterisk) in the corneal epithelium at 24 month. (For interpretation of the references to colour in this figure legend, the reader is referred to the Web version of this article.)

The reduced numbers of axons present in the 24-month-old mouse cornea could be secondary to a number of factors. It could be caused by increased turnover and phagocytosis by the corneal epithelial cells of older, shed axon fragments, reduced expression of mRNAs for proteins that regulate axon extension and targeting, or increased expression of proteins that repel axons. To study the mechanism underlying the loss of axons in older mice, corneal epithelium of 6–8 wk and 24-month-old mice was harvested by debridement and used to isolate RNA. QPCR studies were performed using primers for mRNAs whose expression was associated with increased phagocytosis and autophagy as well as primers for mRNAs associated with axon targeting and extension. Data are presented in Fig. 6 after normalization against mRNA expression in 6–8 wk mice. We found no significant difference in expression at 24 months compared to 6–8 wk for several mRNAs that are up-regulated in cells undergoing increased phagocytosis and autophagy including Beclin1, LC3, LAMP1, and LAMP2. By contrast, among the mRNAs assessed that mediate axon targeting, 4 that positively regulate axon targeting were reduced in expression by 24 months including netrin1 and it's receptors DCC, Unc5b and Efna5. Repulsive guidance molecule A (Rgma) was increased in the corneal epithelium by 24 months. Taken together, these data indicate that aging leads to reduced numbers of intraepithelial corneal nerves and reduced sensitivity in part due to reduced expression in cornea epithelial cells of proteins that induce axon growth and increased expression of proteins that repel axon growth. While there are fewer axons, those that persist retain their ability to target apically.

## 4. Discussion

### 4.1. Relative to vehicle alone, topical MMC treatment does not impact ICN density significantly after initiation of DS

Mitomycin C (MMC) is an antibiotic that induces DNA strand breaks and crosslinks in bacteria (Lown et al., 1976; Tomasz, 1995); in mammalian cells and tissues, it induces cell cycle arrest and reduces scar formation and is used clinically as a chemotherapeutic for cancer patients (Bachur et al., 1979). In ophthalmology, MMC is used to reduce scarring during glaucoma and refractive surgery (Chen and Chang, 2010; Majmudar et al., 2015; Santhiago et al., 2012). Axon density is lowest d10 after DS is initiated in corneas treated at d1 and d3 with vehicle. The difference seen in axon density between vehicle and MMC treated corneas at d5 and d10 is similar to that seen in corneas subjected to DS alone. These experiments indicate that topically applied MMC does not reduce axon density in mice subjected to acute DS. In addition, MMC treatment did not prevent the loss of axon density seen when mice are exposed to acute DS. Although experiments performed on debridement wounded mice indicate that MMC stabilizes axons by increasing adhesion between axons and ECM secreted by corneal epithelial cells (Pal-Ghosh et al., 2016), MMC treatment does not prevent axonal loss due to DS.

### 4.2. By 5 days, acute desiccating stress reduces axon density, thickness, and apical extension of the intraepithelial nerve terminals

The corneal epithelial basal cells maintain the intraepithelial corneal nerves (ICNs) and act as surrogate glial cells to stabilize both the subbasal nerves (SBNs) and the intraepithelial nerve terminals (INTs) that extend towards the apical squames (Stepp et al., 2017). Here we show that acute DS impairs the stability, morphology, and ability of the corneal sensory nerves and their terminals to extend apically and/or to be maintained at apical sites within the corneal epithelium. Since axons closest to the apical surface are more responsive to cold and pain sensations than those that terminate within the subbasal nerve layer, fewer apical terminals would be expected to reduce corneal sensitivity. Corneal touch threshold assessments confirm that corneal sensation is impaired 3d after induction of DS; this is before there is a significant loss of axon density. INT apical targeting is significantly reduced by day 3 but remains 75–80% that seen in controls. The model of acute DS used here has been shown previously to induce loss of barrier function and inflammation within 3–5 days (de Paiva et al., 2006a; 2009). Penetration of the corneal epithelium by proteins from the tear film and/or cytokines released by immune cells may impair axon function and initiate a reduction in axon density over time.

### 4.3. Age related changes are seen in the intraepithelial corneal nerves and in mRNA expression of aged corneal epithelial cells

Between 2 and 24 months of age, axon thickness and nerve terminal apical extension remain the same despite significant loss of axon density. While nerve terminals are present near the apical aspect of the corneal epithelium, corneal sensitivity at 24 months of age is either undetectable or low.

The disappearance of the majority of the sensory axons in the cornea by 24 months of age reported in our study is consistent with an earlier study (Reichard et al., 2016); but extends those data to show that corneal touch sensation is also reduced while apical axon extension is not affected by aging. The nerve terminals that targeted to the apical–most cell layers in the majority of corneas failed to initiate a blink in response in the corneal touch assay. Paradoxically, reduced corneal touch sensation in patients with chronic aqueous tear deficiency is often associated with increased irritation symptoms (Rahman et al., 2015). Belmonte et al. (2015) have shown that nerve loss or damage associated with chronic dry eye can be accompanied by increased spontaneous nociceptor activity and neuropathic pain. Additional studies are needed to determine the mechanism leading to the loss of corneal touch sensation in acute and chronic dry eye.

QPCR was performed to quantify RNAs implicated in the growth, inflammation, and/or phagocytosis of axons. We find that at 24 months, Ntn1, DCC, Unc5b, and Efna5 are all significantly reduced relative to 2-month-old mice and Rgma is increased. By contrast, levels of several RNAs involved in phagocytosis and autophagy are not altered.

Netrins (Ntns) are mammalian homologs of UNC6, an ECM protein containing laminin motifs first identified in C. elegans; netrin receptors, referred to as “dependence receptors”, include several integrins, Dcc, Unc5B, and Neo1 (Mehlen et al., 2011). Ntn1 and Unc5B are expressed by corneal epithelial, and stromal cells (Han et al., 2012). In response to corneal debridement injuries that lead to development of recurrent erosions, expression of Ntn1, Unc5b, DCC, Efna4, Efna5, and Rgma mRNAs within corneal epithelial cells change over time as erosions form and ICN retraction is observed (Pajoohesh-Ganji et al., 2015).

Rgma induces retraction of retinal axons (Monnier et al., 2002) and it's expression was increased in 24-month-old corneal epithelium. Increased Rgma mRNA expression may reduce nerve terminal apical extension. Although reduced expression of Efna4 in 24 month-old mice should favor axon extension apically (Nikolopoulos and Giancotti, 2005), Efna5 expression is retained at levels similar to those seen in 2-month-old mice.

Neuroinflammation impacts development of small fiber neuropathy and alters sensory axon morphology. McClellan et al. (2014) have shown that between 9 and 24 months of age, autoreactive, pathogenic CD4+T cells accumulate in the conjunctivas and lacrimal glands of C57BL/6 mice. Adoptive transfer studies have shown that young RAG1 KO mice injected with CD4+T cells isolated from 24 month-old-mice show loss of goblet cell density in their conjunctivas; whereas the conjunctivas of mice injected with CD4+T cells isolated from young mice retained their goblet cells. When we assessed expression of mRNAs for proteins that mediate phagocytosis of axon fragments in 2-and 24-month-old mice, none were increased in expression with aging making it unlikely that older axons are being degraded at increased rates.

Taken together with the data obtained on mRNAs for axon targeting, these results suggest that older corneas have fewer axons because corneal epithelial cells do not produce the proteins that enhance axon targeting (Netrin1, DCC, Unc5b, and Efna5) at high enough concentrations and/or produce proteins that inhibit axon targeting (Rgma) at higher levels leading to reduced axon density. It is not clear whether reduced corneal epithelial cell proliferation in older corneas is caused by the loss of sensory nerves, increase in immune cells seen in these corneas (McClellan et al., 2014), or to other factors. Studies by Cavanagh and Colley (1982) and Garcia-Hirschfeld et al. (1994) showed that factors released by sympathetic and parasympathetic nerves can regulate corneal epithelial cell proliferation. Additional studies assessing whether sympathetic nerves play a role in the loss of sensory nerves with aging and the reduced rate of corneal epithelial cell proliferation are indicated.

## Summary

Here we show that axon density is reduced in corneas of mice subjected to acute DS as well as in older mice previously shown to have chronic dry eye. Changes in sensory axon morphology and numbers after initiation of acute DS and with aging are accompanied by loss of corneal touch threshold.

## Supplementary Material

supplement
